# Supplementary material for: Characterization of viral pathogens associated with symptomatic upper respiratory tract infection in adults during a low COVID-19 transmission period
Source: PeerJ. 2023 Mar 13;11:e15008. doi: 10.7717/peerj.15008 (PMC10022499; doi:10.7717/peerj.15008)
Supplement: Supplemental Information 1 [file peerj-11-15008-s001.docx]

**APPENDIX**

**Characterization of Viral Pathogens Associated with Symptomatic Upper Respiratory Tract Infection in Adults during a Low COVID-19 Transmission Period.**

Sandybayev N.T.^1*^, Beloussov V.Yu.^1,2^, Strochkov V.M.^1^, Solomadin M.V.^3^, Granica J.^2^, Yegorov S.^4,5*^

**Supplementary Table 1:** Multiplex PCR panel characteristics. IC= internal control.

| PCR mix | Channel | | |
| --- | --- | --- | --- |
|  | green | yellow | orange |
| hRSv - hMpv | IC | hRSv | hMpv |
| hAdv - hBov | IC | hBov | hAdv |
| hRv | IC | – | hRv |
| hPiv 1/3 | IC | hPiv3 | hPiv1 |
| hPiv 2/4 | IC | hPiv2 | hPiv4 |
| hCov | IC | NL-63, 229E | HKU-1, ОС 43 |

**Supplementary Table 2:** Representative amplification plots from the multiplex PCR testing experiment.

| **Target** | **Sample #** | **Amplification plot** |
| --- | --- | --- |
| hAdv | #7 | 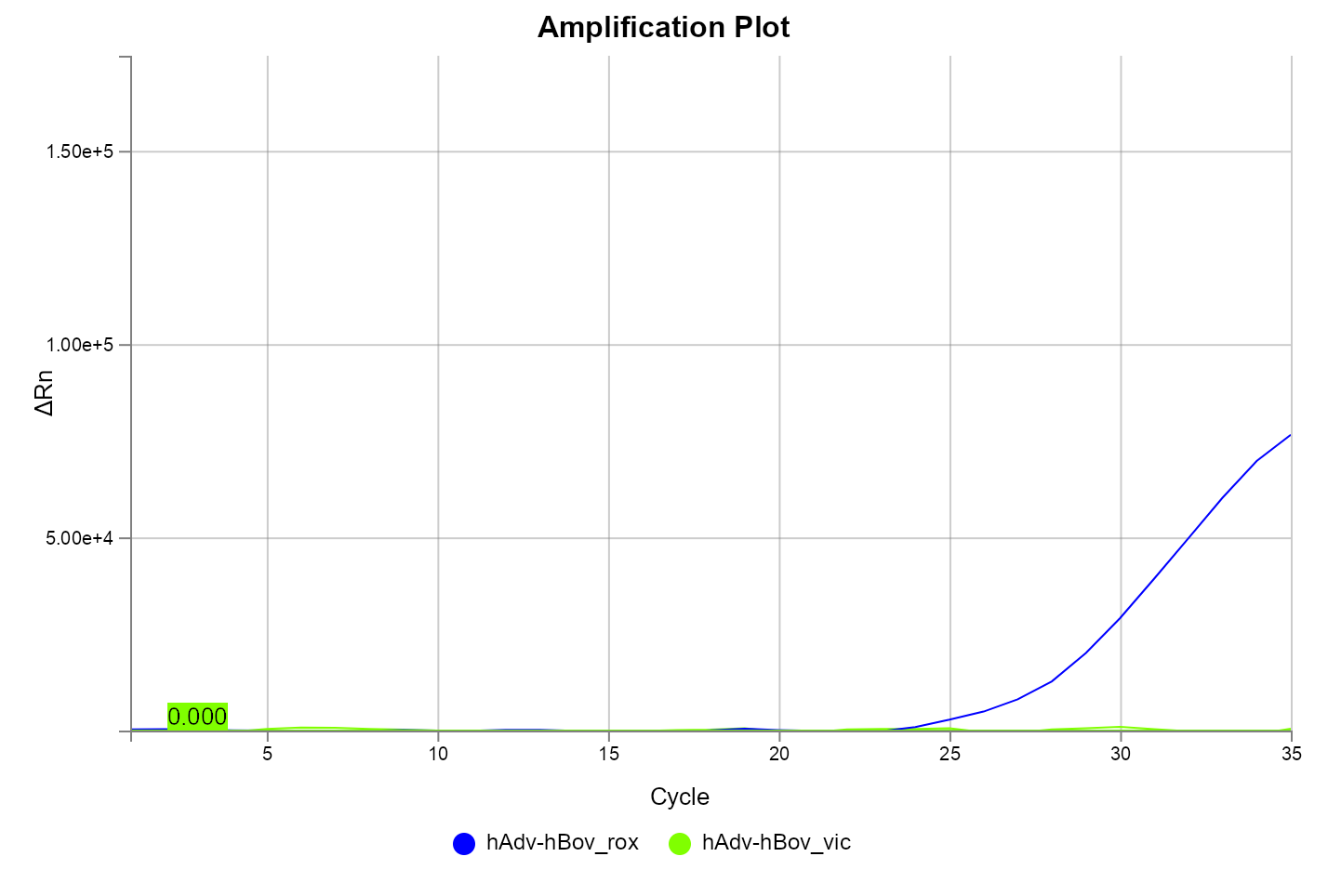 |
| hCoV ОС43/HKU-1 | #7 | 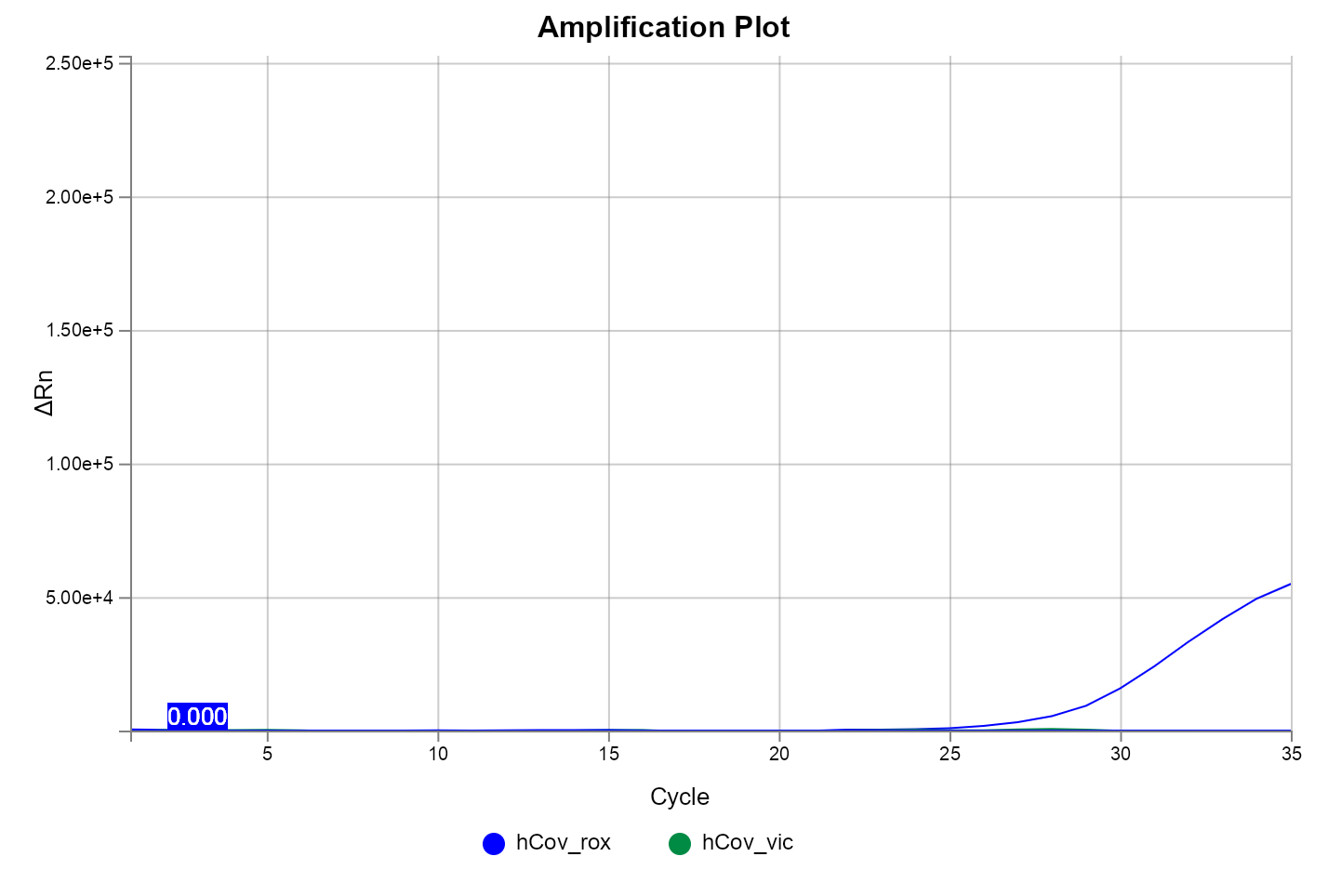 |
| hPIV | #13 | 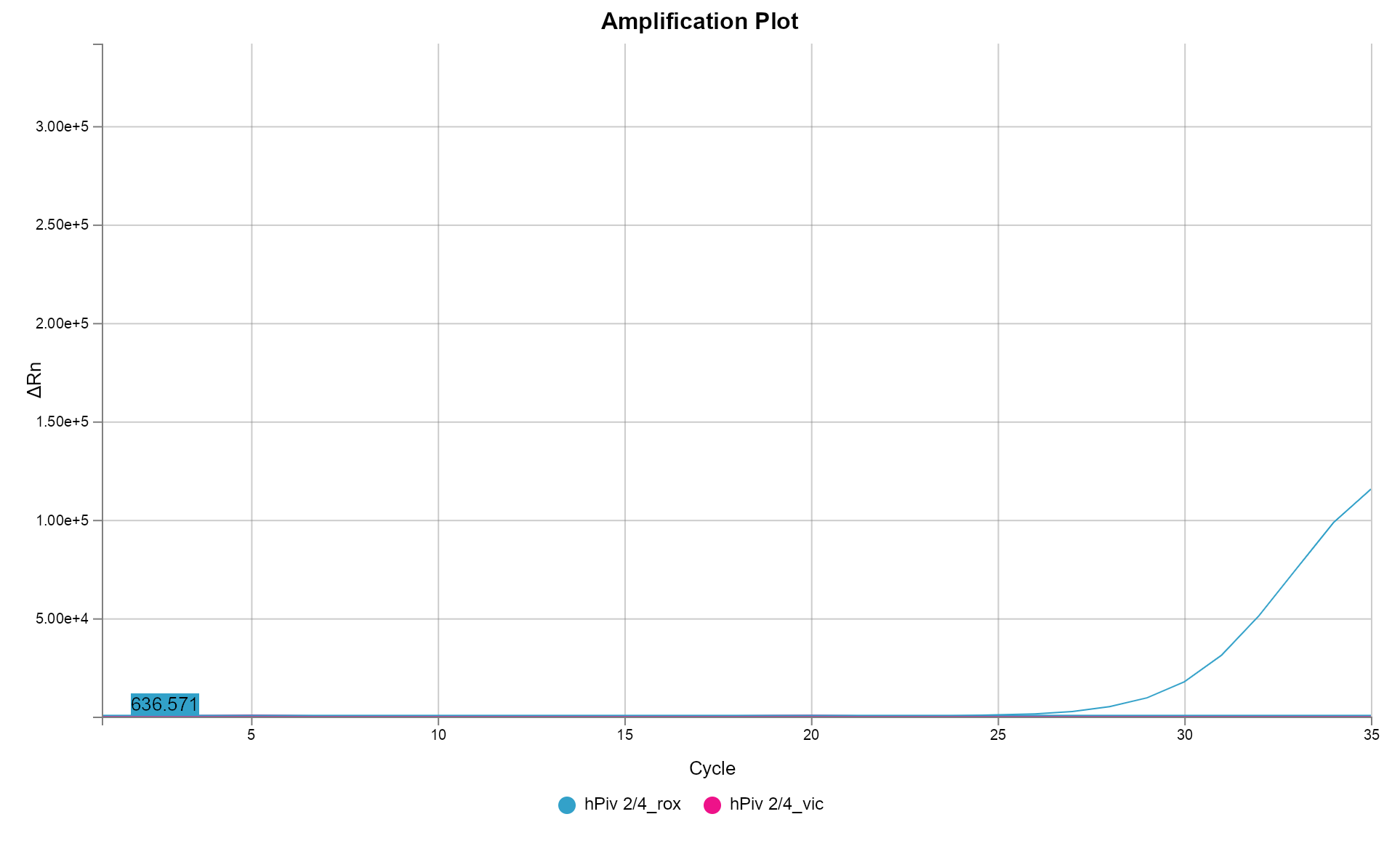 |
| hRV | #75 | ^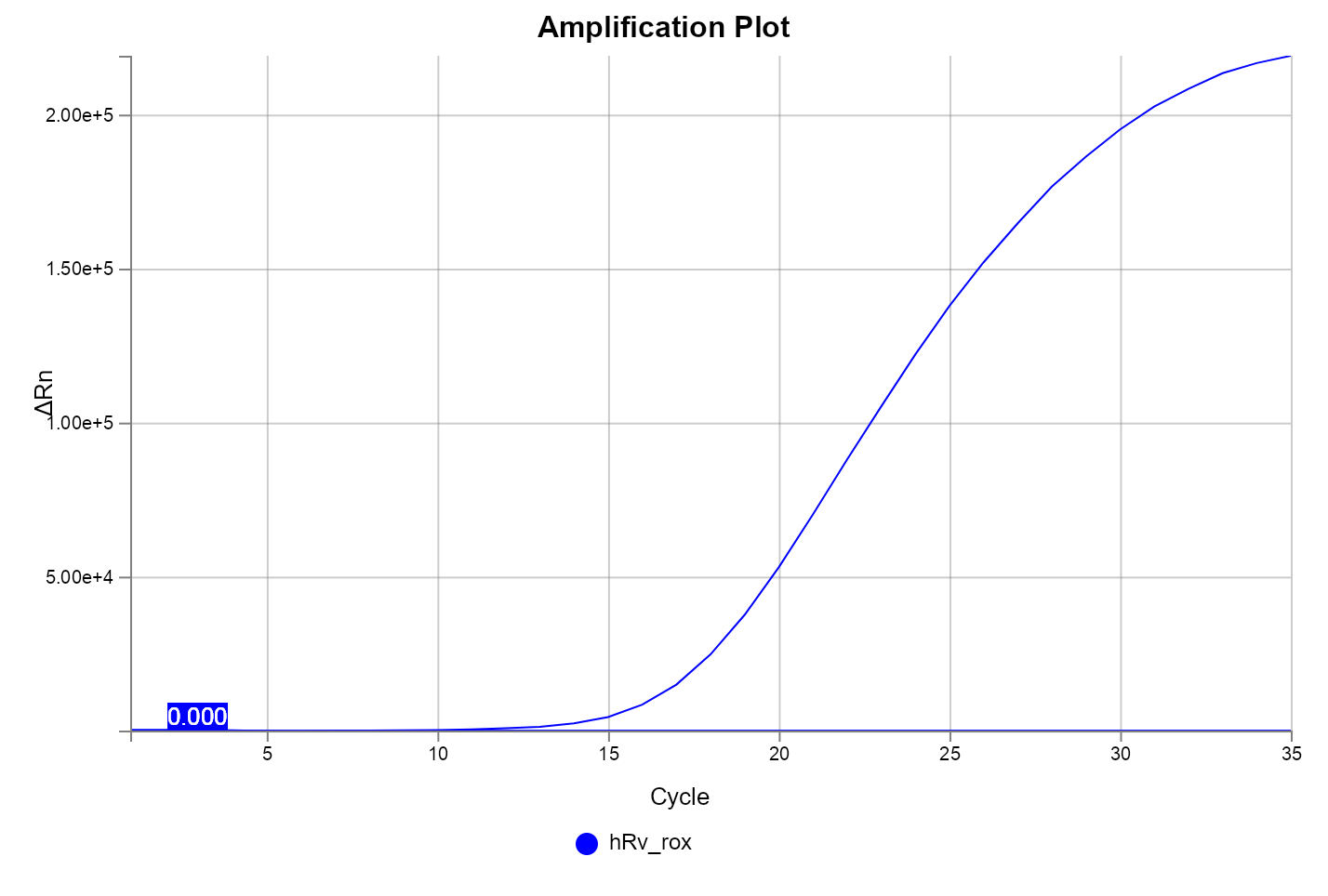^ |
